# Palliative Luminal Treatment of Colorectal Cancer Using Endoscopic Calcium-Electroporation: First Case Series from United Kingdom

**DOI:** 10.3390/jcm14124138

**Published:** 2025-06-11

**Authors:** Ademola Adeyeye, Olaolu Olabintan, Homira Ayubi, Hao Gao, Aman Saini, Andrew Emmanuel, Bu’Hussain Hayee, Amyn Haji

**Affiliations:** King’s College Hospital, Denmark Hill, London SE5 9RS, UK; olaolu.olabintan@nhs.net (O.O.); h.ayubi@nhs.net (H.A.); hao.gao1@nhs.net (H.G.); aman.saini4@nhs.net (A.S.); aemmanuel@nhs.net (A.E.); b.hayee@nhs.net (B.H.); amynhaji@nhs.net (A.H.)

**Keywords:** colorectal cancer, palliation, endoscopy, calcium electroporation, non-thermal ablation, pulsed field ablation

## Abstract

**Background/Objectives:** Colorectal cancer (CRC) is the most common gastrointestinal (GI) malignancy, the second leading cause of cancer-related mortality, and the third most prevalent tumor. Around 20% of cases are metastatic or inoperable at diagnosis, often requiring palliative treatment, which may not be feasible in frail patients. Calcium-electroporation, a less invasive alternative, induces cell death via apoptosis, necrosis, and pyroptosis. This study is the first in the United Kingdom to evaluate the efficacy and safety of endoscopic calcium-electroporation in palliating distal CRC. **Methods**: Frail patients with inoperable left-sided CRC were included. The diagnosis and staging followed standard guidelines, while frailty was assessed using the performance status (PFS), Charlson comorbidity index (CCI), and American Society of Anesthesiologists (ASA) score. Calcium electroporation was performed via a flexible endoscopy usually under sedation, with symptom relief, quality of life (QoL), survival, and adverse events (AE) monitored. **Results:** Sixteen patients (median age 84.5) underwent 36 treatments with electroporation over 28 months (November 2022 to March 2025). The incidence of common symptoms was rectal bleeding (75%), constipation (25%), and pain (75%). Nine patients had metastases and three had failed conventional treatments. Symptomatic relief and an improved QoL occurred in 86.7%, with transfusion/iron infusion needs reduced by 91.7%. The median cancer-specific survival was 10 months, with a 94% survival rate. No device-related AE was recorded. One patient died after 11 months due to disease progression while two patients passed away from other medical conditions. **Conclusions**: Endoscopic calcium electroporation is a safe, palliative option effective for tumor reduction and symptomatic relief in frail CRC patients unfit for conventional therapies.

## 1. Introduction

CRC is the third most prevalent malignancy worldwide and the second leading cause of cancer-related mortality, accounting for approximately 10% of all cancer deaths globally [1,2]. In the United Kingdom (UK), CRC affects over 44,000 individuals annually, with over 16,000 deaths attributed to the disease each year [3]. Despite advances in early detection and treatment, around 20% of patients present with metastatic or inoperable disease at the time of diagnosis, necessitating palliative interventions to alleviate symptoms and improve QoL [4].

For patients with advanced CRC, particularly those who are frail or have significant comorbidities, conventional oncologic treatments such as chemotherapy, radiotherapy, and surgery may be poorly tolerated or contraindicated. Surgical palliation, including colostomy formation or tumor debulking, carries substantial perioperative risks, especially in elderly patients with high ASA scores. Chemotherapy and radiotherapy, while effective in reducing the tumor burden, often result in severe systemic toxicity, further compromising patient well-being. Endoscopic palliation, including argon plasma coagulation (APC) and stenting, offers alternatives for symptom relief but may have a limited efficacy, particularly for some bleeding ulcerated lesions [5] or locally infiltrative tumors distal to the recto-sigmoid junction [6].

Given these challenges, there is a pressing need for innovative, minimally invasive palliative therapies that can improve outcomes in this vulnerable patient population.

Calcium-electroporation (Ca-EP) is an emerging, non-thermal tumor ablation technique that utilizes short, high-voltage electric pulses to transiently permeabilize cell membranes, allowing for the influx of supra-physiological levels of calcium ions [7]. The resulting intracellular calcium overload triggers mitochondrial dysfunction, leading to rapid energy depletion and subsequent cell death through apoptosis, necrosis, and pyroptosis [8]. Unlike chemotherapeutic agents, Ca-EP exploits a fundamental biochemical vulnerability of cancer cells—an impaired ability to regulate calcium homeostasis—while sparing surrounding healthy tissues [7]. The mechanism of the Ca-EP-induced tumor cell death, particularly its immunogenic effects [9], is an area of additional interest in the scientific community. Unlike thermal ablation techniques, which cause coagulative necrosis with limited immunogenicity, the potential of electroporation to induce a form of immunogenic cell death that promotes local immune activation is currently being investigated.

The therapeutic potential of Ca-EP has been demonstrated in preclinical and clinical studies, primarily in cutaneous malignancies and soft tissue sarcomas. Recent studies evaluating Ca-EP for the treatment of cutaneous metastases have shown significant tumor regression with minimal adverse effects [10]. Other studies have explored its application in pancreatic and head-and-neck cancers, suggesting a broader oncologic utility [11,12], while teams in Ireland and Denmark have explored Ca-EP for the palliative treatment of gastrointestinal cancer [13,14,15,16,17,18]. CRC, particularly in its advanced stages, presents with distressing symptoms such as rectal bleeding, pain, bowel obstruction, and tenesmus [4]. For frail patients who are unsuitable for surgery or systemic therapy, endoscopic palliation provides a valuable means of symptom control while avoiding the morbidity associated with more invasive interventions. The adaptation of Ca-EP for endoscopic delivery in CRC is a logical extension of its therapeutic potential, offering a targeted approach to tumor ablation while preserving bowel integrity. Despite being considered innovative, Ca-EP is not an experimental technique and is known to be used clinically for the treatment of GI cancer in hospitals across the UK [19], Spain [20,21], Italy [22], and Germany [23].

However, there is a paucity of published data on endoscopic Ca-EP in CRC. This study aims to assess its safety, feasibility, and palliative efficacy in frail patients with inoperable left-sided colon and rectal cancer. By assessing symptom relief, QoL improvements, and survival outcomes, we seek to establish a foundation for future research into the integration of Ca-EP as a standard palliative modality for CRC.

## 2. Materials and Methods

### 2.1. Setting

Patients included in this case series were treated at King’s College Hospital London, a major referral center in the UK, between 2022 and 2024. Those treated were frail patients diagnosed with inoperable, locally symptomatic left-sided CRC undergoing endoscopic Ca-EP as a palliative intervention in a real-world clinical setting. Before treatment, all patients consented to the use of their data to be used in current or future studies. The protocol was approved by the New Clinical Procedures Committee of King’s College Hospital (KCH) for ethics and endorsed by the multidisciplinary teams of the South East London Cancer Alliance (SELCA) Network. Ethics was waived as the review was considered to be an audit of clinical practice in line with the guidelines for the KCH National Health Service (NHS) trust.

### 2.2. Eligibility

Adult patients discussed by the multidisciplinary team (MDT) were considered for the study if they met the following criteria: histologically confirmed CRC with a lumen passable by an endoscope; symptomatic local disease, including tumor-related bleeding, pain, tenesmus, or subacute obstruction; unsuitable for surgical resection or conventional oncologic treatments (chemotherapy and radiotherapy) due to frailty, comorbidities, or patient preference; Eastern Cooperative Oncology Group (ECOG) performance status ≥ 3; ASA score of ≤5; and CCI ≥ 10. Patients were excluded from the study if they met any of the following criteria: pregnant or breastfeeding; had implanted colorectal stents; had severe coagulation disorders (INR > 2, platelet count < 50,000 per microliter of blood); had highly inflamed colonic mucosa; and had cardiac devices (e.g., pacemakers, and implantable defibrillators) that could not be deactivated for more than 30 min.

Once patient eligibility was determined following MDT discussion, informed consent was obtained before enrolment. The MDT, which also included a patient advocate, provided governance and oversight of the care of these potentially vulnerable patients on a case-by-case basis.

### 2.3. Pre-Procedure Evaluation

All patients underwent a structured preoperative assessment, including full medical history (emphasizing comorbidities, prior oncologic treatments, and contraindications to lower GI endoscopy); physical examination, including digital rectal examination where necessary; laboratory investigations, including full blood count (usually within one week of procedure); coagulation profile (usually within 24 h of procedure); and renal function tests. Bowel preparation was initiated as per institutional colonoscopy guidelines and included oral laxatives ± rectal enemas on arrival at the endoscopy suite. On the day of the procedure, intravenous access (IV) was established for sedation and fluid administration if required. Sedation requirements were determined on an individual basis and were sometimes omitted at the request of the patients. The typical medications given were fentanyl with or without midazolam. General anesthesia was not used and antispasmodic agents (e.g., hyoscine butylbromide) were used selectively.

### 2.4. Endoscopic Calcium Electroporation Protocol

Ca-EP was performed using the Conformité Europénne (CE)-marked and Medical Device Directive (MDD)-certified (2027/MDD) ePORE^®^ electroporation generator and single-use CE-marked EndoVE^®^ probe (Mirai Medical, Galway, Ireland). The procedure was carried out in an outpatient setting under sedation, following standard endoscopy protocols. The EndoVE^®^ probe was attached to the ePORE^®^ generator using an extension lead that was pre-programmed with the parameters required to deliver the pulsed electric fields required for the electroporation of tissue. The EndoVE^®^ probe was also connected to a vacuum, which facilitated the tumor tissue being held in place within the chamber of the probe, for the duration of pulse delivery.

**Figure 1:**
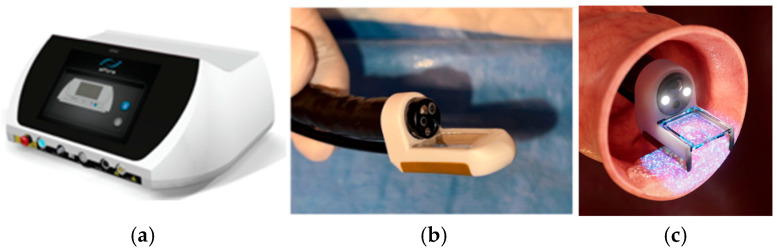
(**a**) The ePORE^®^ generator; (**b**) the EndoVE^®^ electrode; and (**c**) an illustration of the application of pulsed electric fields (electroporation) using EndoVE^®^.

Equipment and Materials: ePORE^®^ generator (Figure 1) with pre-programmed extension lead; EndoVE^®^ probe (Figure 1) with vacuum-assisted tumor engagement; endoscopic injection with vacuum-assisted tumor engagement; endoscopic injection needle (4 mm) and 10 mL syringe; 10% calcium gluconate ampoules (preferred over calcium chloride for its superior hemostatic properties and reduced tissue electrical impedance); high-suction vacuum system (>400 mmHg); and standard flexible gastrointestinal endoscope with a tip diameter <11 mm (e.g., gastroscope or pediatric colonoscope) to conform with the size of the EndoVE^®^ probe.

Procedural Steps:

Patient Positioning and Monitoring: Patients were positioned in the left lateral decubitus position (but modified as necessary). Vital signs, including oxygen saturation and heart rate, were continuously monitored and supplemental oxygen via nasal cannula was provided in accordance with British Society of Gastroenterology (BSG) guidelines.

Initial Endoscopic Assessment: Endoscopic assessment was performed using a lubricated gastrointestinal endoscope (10–11 mm diameter) to determine the site, size, and other morphological characteristics of the tumor.

Calcium Injection: Calcium was injected into the tumor before each application of the pulsed electric fields. This was achieved by introducing the endoscopic injection needled through the working channel of the endoscope and injecting the calcium gluconate (10%; ≤10 mL per session). Injection volume was determined based on tumor size and vascularity, ensuring even distribution.

Electroporation Delivery: The EndoVE^®^ probe was mounted on the tip of the endoscope and advanced to the tumor site. The probe was activated with high-suction vacuum engagement to maximize tumor contact. Pulsed electric fields (1 cm penetration depth, 2 cm^3^ surface area coverage) were delivered. The pulses were applied after each calcium injection and were followed by repeated application as required until all available surface area was treated. Key procedural parameters were recorded, including tumor location and size; estimated percentage of tumor surface treated; the volume of calcium gluconate injected; the number of pulses delivered; maximum applied current; and lowest tissue impedance.

Completion and Withdrawal: The probe was disengaged by turning off the vacuum, and the endoscope was carefully withdrawn. Patients were observed for at least 30 min post-procedure before discharge.

Post-Procedure Care and Follow-Up: Patients were monitored according to standard endoscopy post-procedure protocols. All patients were discharged on the day of treatment; however, there was a provision for overnight observation if clinically indicated, due to the high-risk profile of the patients. Routine outpatient follow-up was scheduled within 4 weeks for symptom assessment and repeat imaging if necessary. Subsequent Ca-EP sessions were performed as required and at varying intervals, depending on symptom recurrence and clinical response.

Outcome Measures: Primary endpoints were symptom relief (e.g., reduction in rectal bleeding, pain, and constipation); QOL assessed via the 12-Item Short Form Survey (SF-12) Questionnaire for patient-reported outcomes; safety, including the incidence of treatment-related adverse events; and tumor response. Secondary endpoints were reduction in blood transfusion requirements for hemorrhagic tumors and overall survival (OS) at 6 and 12 months post-treatment. The data was prospectively collected and retrospectively analyzed.

Statistical Analysis: Descriptive statistics were used to summarize baseline demographics, procedural data, and clinical outcomes. Symptom relief was evaluated using patient-reported measures and clinician assessment at follow-up visits. Survival analysis was performed using Kaplan–Meier methods.

## 3. Results

### 3.1. Patient Demographics and Clinical Characteristics

Sixteen patients (ten male and six female) were enrolled in the study and underwent a total of 36 endoscopic Ca-EP sessions over 28 months (2022–2025). The median age was 84.5 years (range: 63–92 years), and all patients were classified as frail based on clinical assessments. The majority of patients (56%, *n* = 9) had metastatic disease, while 25% (*n* = 4) had previously failed conventional oncologic treatments (Table 1).

All patients had a poor performance status, with an ECOG performance status of ≥3, an ASA score of 5, and an average CCI of 15, indicating a 0% estimated 10-year survival. The main indications for the Ca-EP treatment were the symptomatic relief for rectal bleeding (75%, *n* = 12), constipation (25%, *n* = 4), and pain (68.75%, *n* = 11).

### 3.2. Primary Endpoints

#### 3.2.1. Safety Assessment

No intra- or post-operative serious adverse events (SAEs) or device-related adverse events (AEs) were reported. There were no cases of colonic perforation, severe bleeding requiring intervention, infection, or post-procedural complications requiring hospitalization. Minor adverse events unrelated to the device included mild discomfort or transient rectal irritation in three patients (19%), which resolved within 24–48 h. The patient-reported self-limiting rectal bleeding occurred in two patients (12%) on anticoagulants and antiplatelet medication following the procedure, and this did not require an additional treatment. One patient (Patient 14) experienced an episode of labile hypotension related to the effects of anti-hypertensive medication immediately after the first procedure under sedation. This phenomenon did not reoccur subsequently after the modification of the relevant medications.

Three of the sixteen patients (19%) have died; one as a result of metastatic disease and two for reasons unattributed to their cancer. None of the reported deaths were device-related.

#### 3.2.2. Symptomatic Response

All patients who were symptomatic resulting from their disease before the treatment experienced a symptomatic response (Table 2). Bleeding/anemia (75%) and pain (68.75%) were the most common symptoms reported, followed by constipation. Of the 12 patients who reported bleeding/anemia before treatment, 75% (*n* = 9) had a permanent cessation of bleeding or a rise in hemoglobin levels which were sustained within a normal range following the treatment; though, one patient required two treatments to achieve a permanent cessation. The bleeding cessation has been sustained for between 3 and 30 months. Approximately 16.66% (n = 2) had a temporary cessation of bleeding, where the approximate duration between the treatment and bleeding recurrence ranged from between 4 and 9 months, and the follow-up for one patient (6.25%) was pending. All patients who had a recurrence of bleeding received an additional Ca-EP treatment for symptom control. Of the twelve patients who presented with bleeding/anemia, two had previously received radiotherapy. Of these two patients, one (Patient 6) had a complete symptomatic response; she was asymptomatic with stable hemoglobin levels 10 months after a single Ca-EP treatment, and the second (Patient 13) also reported a symptomatic response for bleeding following a single Ca-EP treatment.

Eleven patients (68.75%) also reported disease-related pain before their initial treatment, and all reported a sustained improvement of pain after a single treatment, as evidenced by reduced analgesic requirements. Of the four patients who experienced constipation as a symptom of their disease, 75% (*n* = 3) had a complete symptomatic response (range 2 months–18 months). One patient reported the use of occasional laxatives. Other symptoms that responded to the Ca-EP treatment were tenesmus (*n* = 1), changes in movements (*n* = 2), and bloating/flatulence (*n* = 1). Two patients experienced diarrhea before the treatment, which improved following a single Ca-EP treatment. Two patients (11 and 13) reported a distressing mucoid discharge. Patient 11 reported a 50% reduction in mucus discharge after his initial treatment and a complete cessation of symptoms following a second treatment, one month later. Patient 13 reported a complete cessation of symptoms following one treatment. One patient had incontinence before treatment, attributed to co-existing Multiple Sclerosis, which has persisted following the Ca-EP therapy.

#### 3.2.3. Quality of Life Assessment Response

The impact of the treatment on patients’ QOL was assessed using the SF-12 Questionnaire which evaluates physical and mental domains. The questionnaire was administered before the initial treatment and within the first week following the Ca-EP. Twelve patients were able to respond to the questionnaire. Three patients were unable to give a response due to dementia, while one patient (Patient 12) declined. Ten patients of the twelve respondents reported improvements in their QOL. The average change in the physical component summary domain was +23, while that of the mental component summary was +11.5. These values suggest improvements in both physical and mental well-being. Two patients reported no change in the SF-12 survey.

#### 3.2.4. Tumor Response

Eight patients received a single Ca-EP treatment, and eight patients had multiple treatments (between two and five). The number of treatments per patient was determined by the clinical team based on factors such as the patient’s suitability and willingness to undergo multiple treatments, tumor response, and symptomatic relief from previous treatment(s). Approximately 43.75% of patients had cancer of the sigmoid colon (*n* = 7). Rectal cancer was the next most common indication (37.5%; *n* = 6), followed by recto-sigmoid (12.5%; *n* = 2) and descending colon cancer (6.25%; *n* = 1). The number of treatments per indication translated totaled 19 sigmoid colon treatments (53%), followed by 12 rectal (33%), 4 recto-sigmoid (11%), and 1 descending colon (3%).

**Figure 2:**
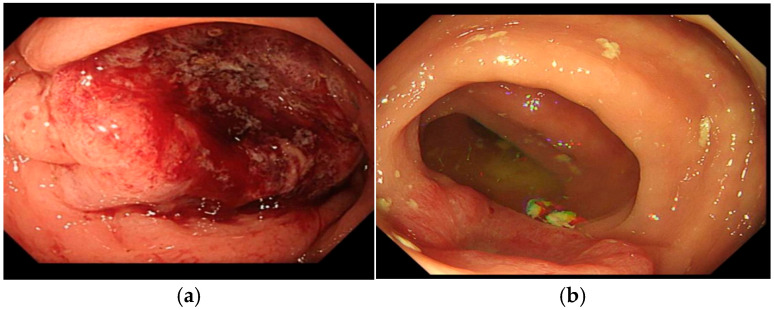
(**a**) Patient 5; an image of the pre-treatment tumor; (**b**) Patient 5; an image of the tumor 5 months after a single session of Ca-EP. This patient went on to have multiple Ca-EP treatments.

In the 75% of patients who returned for endoscopic assessments (*n* = 11) or for whom a CT confirmation of the tumor response was available (n = 1), the overall tumor response rate was 83.3% (*n* = 10) (Table 3). One patient (Patient 7) had a complete clinical response after one treatment, based on CT reporting. This patient did not return for an endoscopic review. Approximately 56% (*n* = 9) of patients experienced a sustained tumor response. However, Patient 9’s tumor increased after the initial treatment and decreased following subsequent treatments. For one patient (Patient 3), the response was transitory, with a reduction in tumor size following the first Ca-EP treatment, but an increase following subsequent treatments, indicating disease progression. For Patient 4, who received multiple treatments for symptomatic bleeding, there was a plateau in the tumor response following the fourth treatment. Sixteen months after initial treatment, her symptoms indicated disease progression. At the time of the report writing this patient is awaiting an endoscopic assessment. Three patients (19%) reported symptomatic relief and chose not to return for the endoscopic assessment; thus, the tumor response could not be assessed. Stable disease was reported for two patients (13%), each of whom received a single Ca-EP treatment, and one patient (6%) declined to return for further treatments or follow-ups. An example of endoscopic response is shown in Figure 2.

### 3.3. Secondary Endpoints

#### 3.3.1. Overall Survival (OS)

At the time of reporting, the overall response rate was 81.3% (*n* = 13), and the per-patient median survival was 10 months (1–29). Ten patients have reached the 6-month follow-up milestone, at which point all (100%) were alive. The OS at 12 months was 80%. Patients 1–5 have reached the 12-month follow-up milestone, at which time four were alive while one patient (Patient 2) who received a single Ca-EP treatment died 11 months later, due to metastatic disease. Patient 8 died 9 months after the treatment and Patient 3 died outside of the 12-month follow-up window. The cause of death was related to their comorbidities. Patients 11–16 had their first Ca-EP treatment in 2025, and they have not yet reached the OS reporting milestones.

#### 3.3.2. Reduction in Blood Transfusion and Iron Requirements

Twelve patients required iron infusions and/or blood transfusions on account of symptomatic anemia prior to the Ca-EP treatment. Overall, 91.7% (*n* = 11) of patients no longer required treatment for anemia following the Ca-EP therapy. Following the treatment, one patient (Patient 5) required an iron infusion 8 weeks after his initial Ca-EP treatment. He went on to have additional Ca-EP treatments and had no further bleeding symptoms or requirements for infusions.

## 4. Discussion

This study presents the first UK case series evaluating endoscopic Ca-EP as a palliative treatment for frail patients with inoperable CRC. The recent data suggests that the number of such patients is on the rise [24,25], and this poses a challenge to clinicians and MDTs when determining the best course of individualized action, particularly in settings where therapeutic nihilism is the typical norm for these cases. Our study findings suggest that Ca-EP is a safe, well-tolerated, and potentially effective option for symptom relief, particularly in patients who are unsuitable for conventional oncologic therapies. This correlates with findings from international teams who have concluded that Ca-EP is safe and has proven effective for tumor debulking and the palliation of disease-related symptoms [13,14]. The median age of patients treated was 84.5 years, with 94% (*n* = 15) of patients aged 79 or older. These elderly patients are often too frail or comorbid for surgery or are unwilling to undergo surgery due to potential risks and side effects (e.g., colostomy) [4]. A recent, unpublished retrospective review was conducted at KCH of the historical data (2013–2022) for patients with confirmed colorectal cancer who were considered for palliative treatments following an MDT review. The median age of the patients included in the review (*n* = 267) was 74.7 years, and approximately 60% of patients had an ECOG performance status of two at diagnosis [26]. When compared to the data from our study, both the lower median age and better performance status of these patients suggest that those patients may have benefitted from Ca-EP as part of their treatment regimen, had it been available in our setting at the time.

The high rate of symptom relief (86.7%), significant reduction in rectal bleeding, and decrease in blood transfusion as well as iron infusion requirements (91.7%) highlight the potential role of Ca-EP as a valuable addition to the palliative treatment landscape for CRC. These correlate with the findings from Broholm et al., where 90% (10/11) of patients who had disease-related bleeding before treatment reported a cessation of bleeding within 2 days following treatment [15]. Hansen et al. also reported similar findings using bleomycin instead of calcium during electroporation [14]. There is a possibility that better responses would have been seen had IV bleomycin been used in place of calcium gluconate, particularly for patients with extensive, bulky, or bleeding tumors. The significance of this is particularly impactful for patients who require multiple transfusions/infusions before receiving Ca-EP treatments. Patient 5, for example, received four units of blood transfusions in the 4 months between their diagnosis and treatment with Ca-EP. These required two separate in-patient admissions, each lasting 48 h. He also received multiple iron infusions during this time. These infusions require multiple hospital trips (one or two per week). The frequency of transfusions as well as the requirement for inpatient hospitalization for each infusion puts additional financial costs and burdens on an already strained health service. Following the Ca-EP treatment, his GI bleeding subsided, the blood counts remained stable, and there was no need for transfusions after therapy. He had a single dose of an iron infusion and erythropoietin 10 weeks later from anemia due to chronic kidney disease. The positive responsive rate seen in bleeding suggests that endoscopic Ca-EP could help improve patients’ quality of life and reduce the burden such patients pose to the health system.

Chemotherapeutic regimens, such as FOLFIRI and FOLFOX4, are typically used in the treatment of non-frail patients with advanced or metastatic colorectal cancer. These patients are often unsuitable for the patient population in this study, mainly due to systemic toxicity, leading to significant morbidity and potential mortality. The lack of systemic side effects and the relative safety of Ca-EP make it an attractive potential option in scenarios where chemotherapy is not possible. For frail patients with inoperable CRC, standard palliative interventions include systemic therapy, which is often poorly tolerated in these individuals due to significant toxicity. Surgical palliation (diverting colostomy and tumor debulking) is another option for obstructing/bulky tumors but can be associated with high perioperative risks, particularly in elderly patients with multiple comorbidities. Where surgical palliation is considered, Ca-EP can be considered a neoadjuvant treatment for tumor debulking. Brohlom et al. investigated this treatment option in their recent study. The majority (90%; n = 19) of patients in that study had surgery as scheduled, following a Ca-EP treatment, while planned surgery was delayed for two patients [15]. In addition, other endoscopic techniques such as colonic stenting, APC, or laser therapy can be effective in some cases but limited in patients with diffuse or bleeding tumors as well as lesions distal to the recto-sigmoid junction [5,6]. Due to the side effects, recovery times, and impact on patients’ QOL, a recent study suggested that it may be more beneficial for frail, inoperable patients to be referred for best supportive care, instead of standard-of-care interventions such as those outlined above [27]. In such cases, Ca-EP could be a worthwhile adjunct to best supportive care by improving the QOL of these patients, as indicated by the 83.3% of patients surveyed who reported an improvement in their QOL following Ca-EP treatments.

Compared to these approaches, Ca-EP offers several distinct advantages. It is a minimally invasive outpatient-based procedure that is performed under sedation via a flexible endoscopy, avoiding the need for general anesthesia or hospitalization. This is particularly important for frail patients with high ASA or CCI scores who are at a significant risk of perioperative complications. We also noticed sustained symptom control in most patients. While most patients required multiple sessions (mean: 2.1 per patient), the procedure was well tolerated and effectively maintained symptom relief over time. The ability to repeat the procedure every 2–3 months, or more frequently if indicated, provides ongoing palliation without cumulative toxicity. Unlike thermal ablation techniques (e.g., APC or laser therapy), which carry risks of mucosal damage and perforation, Ca-EP spares the surrounding healthy tissue, reducing the likelihood of major complications [7]. Furthermore, the absence of device-related adverse events underscores its safety, making it an ideal option for frail patients who cannot tolerate aggressive interventions. Patient 14 experienced an episode of hypotension immediately following the Ca-EP treatment; however, upon further evaluation, it was determined that this was due to anti-hypertensive medications that the patient was taking. After a review of the medication by a cardiologist, the patient was deemed fit to receive additional Ca-EP, and no hypotension episode was observed in subsequent sessions.

The inclusion of Ca-EP as a treatment modality for these patients offers them treatment options where they may not otherwise have one, as well as the potential to extend their life expectancy and improve their QOL with the palliation of symptoms. Moreover, the treatment with Ca-EP does not preclude patients from being treated with standard interventions (chemotherapy, immunotherapy, targeted therapy, and surgery) if required. Though the scientific and medical communities are still building the volume of evidence required to increase awareness of Ca-EP treatments, it has been shown to be both safe and effective, as demonstrated by this study. The CRC MDT at King’s College Hospital as well as the SELCA Network adjudged that it was worth exploring the safety, efficacy, as well as the feasibility of this therapy in this category of patients, providing governance and oversight during this process. These bodies also have patient advocates as members, and this was important in ethical regulations of this treatment of this potentially vulnerable patient group.

The emerging evidence suggests that treatments with electroporation can stimulate anti-tumor immune responses by inducing immunogenic cell death (ICD) in pancreatic and prostate cancers [28,29,30,31]. The reporting of this phenomenon is most common regarding irreversible electroporation (IRE). However, Broholm et al. plan to explore if Ca-EP can produce similar findings when used in the neo-adjuvant treatment of colorectal cancer [15]. Should they find that the immunogenic effects of Ca-EP mirror those of IRE, future research could explore combination strategies with immune checkpoint inhibitors (ICIs) to enhance systemic anti-tumor immunity and the potential of Ca-EP to induce systemic tumor regression in metastatic CRC [32]. 

### 4.1. Limitations of This Study

Despite the promising findings, this study has important limitations that warrant careful interpretation. The (underpowered) small sample size (*n* = 16), absence of a comparator arm, and descriptive study design limit the strength of causal inferences. Although we observed a median cancer-specific survival of 10 months, the lack of matched controls as well as the relatively short follow precludes definitive statements regarding long-term efficacy. The lack of a comparison with standard endoscopic palliation (e.g., APC or stenting) or best supportive care is also a major limitation. Our inability to establish a longitudinal assessment of the durability of the response symptomatic relief and long-term safety is also important to consider. Additionally, the heterogeneity in the tumor biology and disease stage could be responsible for the variability in tumor response rates.

### 4.2. Future Studies

Future studies should include randomized comparative studies that compare Ca-EP with standard palliative treatments (e.g., APC, stenting, and palliative radiotherapy), stratifying patients based on the tumor location, burden, and molecular profile. Studies comparing Ca-EP with the best supportive care will also help clarify the possibility of a placebo effect.

Larger, multi-center randomized trials are required to assess the outcomes of Ca-EP across larger patient populations to reflect the diversity in patient demographics, including age, frailty, and ethnicity. These studies would serve to evaluate long-term outcomes, including the survival beyond 12 months and potential late toxicities. The possible hypothesis that Ca-EP may have a synergistic role in combination with immunotherapy needs to be proven to determine if it has potential systemic anti-tumor effects. This may be of benefit particularly in patients with microsatellite instability–high (MSI-H) tumors due to an enhanced immune engagement. This will serve as motivation for further translational research and the role of biomarkers in this novel therapy. Tumor biology, technical factors related to the procedure, as well as appropriate time intervals between treatment sessions are potential determinants of cancer responses to Ca-EP.

Health economic studies to assess if there is a cost–benefit in treating patients with Ca-EP when compared with the cost of other modalities (e.g., chemotherapy, radiotherapy), particularly with regard to bleeding control, may also be beneficial.

## 5. Conclusions

Based on the findings of our review, we conclude that Ca-EP is a safe, potentially effective, and repeatable treatment option for frail patients with inoperable CRC who are unsuitable for conventional oncologic therapy. The majority of patients in this study reported an improved quality of life and a sustained improvement in the palliation of disease-related symptoms, such as bleeding, pain, and constipation.

## Figures and Tables

**Table 1:** Patient characteristics, stage of disease, medical history, and prior treatments.

Patient	Sex	Age	Tumor Location	Stage of Disease	ASA Score	ECOG Performance Score	Comorbidities	Previous Treatment
Patient 1	F	92	Sigmoid	Nonmetastatic	5	3	Chronic kidney disease	None
Patient 2	F	88	Sigmoid	Metastatic	5	4	Heart disease, hypercholesteremia, asthma, cataracts	Neoadjuvant CRT, loop colostomy
Patient 3	M	89	Sigmoid	Metastatic	4	4	Osteoporosis, pathological fractures, bed sores, squamous cell carcinoma, actinic keratosis, cardiac arrhythmias on pacemaker and anticoagulants	None
Patient 4	F	86	Rectal	Nonmetastatic	5	4	Alzheimer’s, hypertension, heart disease, stroke (on anticoagulants), asthma, hyperlipidemia	None
Patient 5	M	79	Sigmoid	Nonmetastatic	5	3	Atrial fibrillation, ischemic heart disease, systemic hypertension, pulmonary hypertension, liver cirrhosis, Type 2 diabetes, chronic kidney disease, basal cell carcinoma of the skin, prostate cancer	None
Patient 6	F	86	Rectal	Metastatic	5	3	Ischemic heart disease (on anticoagulants), hyperlipidemia, depression	Radiotherapy
Patient 7	M	92	Descending colon	Metastatic	5	4	Osteoporosis, pathological fractures, postural hypotension (on long-term steroids), pre-diabetes, hiatal hernia, glaucoma	None
Patient 8	M	80	Sigmoid	Metastatic	5	5	Hypertension, deep vein thrombosis, atrial fibrillation (on anticoagulants), pulmonary embolism, heart failure	None
Patient 9	M	84	Sigmoid	Metastatic	4	3	Hypertension, chronic kidney, chronic obstructive lung disease, benign prostate hyperplasia, asthma	None
Patient 10	M	89	Sigmoid	Nonmetastatic	4	3	Type 2 diabetes, hypertension, heart failure, chronic kidney disease	None
Patient 11	M	88	Rectal	Metastatic	4	3	Hypertension, chronic kidney disease, benign prostatic hyperplasia, hyperlipidemia	None
Patient 12	M	87	Rectal	Metastatic	4	3	Hypertension, hyperlipidemia	Chemo- radiotherapy
Patient 13	F	63	Rectal	Metastatic	4	3	Stroke	Surgery, chemotherapy, immunotherapy, radiotherapy
Patient 14	F	85	Rectal	Nonmetastatic	4	3	Pre-diabetes, osteopenia, hypertension, chronic obstructive lung disease, chronic kidney disease	Chemo-radiotherapy
Patient 15	M	81	Sigmoid	Nonmetastatic	4	3	Stroke, hypertension, deep vein thrombosis, atrial fibrillation (on anticoagulants), chronic kidney disease	None
Patient 16	M	86	Recto Sigmoid	Nonmetastatic	4		Multiple Sclerosis with pulmonary embolism and on anticoagulants	None

**Table 2:** Symptomatic response to Ca-EP.

Patient	Presenting Symptoms	Symptomatic Response	Duration of Response at Time of Reporting
Patient 1	Pain, Bleeding, Constipation	Yes	30 months at last follow-up for pain and bleeding. Occasional use of laxatives.
Patient 2	Bleeding, Constipation	Yes	The patient died 11 months after treatment. Asymptomatic for bleeding and constipation for duration.
Patient 3	Change in Movements, pain, Bleeding, Anemia, Fatigue,	Yes	Durable response for change in movement, pain, and fatigue for 8 months. Cessation of bleeding following first treatment for approx. 3 months. Cessation in bleeding following third treatment for 6 months. The patient died 12 months after the first treatment; the cause of death was not device-related.
Patient 4	Constipation, Pain, Bleeding	Yes	Durable response for pain and constipation following first treatment (approx. 1.5 years). Transient response for bleeding between treatments (median interval between visits = 16.75 weeks).
Patient 5	Anemia Requiring Multiple Blood Transfusions and Iron Infusions	Yes	Durable response for anemia to 12 months. The patient has not required a blood transfusion since initial treatment (12 months). The patient required one iron infusion 8 weeks after initial treatment. Likely that this is a result of underlying kidney disease.
Patient 6	Anemia, Pain	Yes	12 months for anemia and pain.
Patient 7	Pain, Bleeding	Yes	12 months for bleeding and pain. Complete response to treatment.
Patient 8	Change in bowels, Loose Stools, Bloating, Flatulence	Yes	Symptomatic response for 2 months. The patient died two months after treatment; unrelated causality.
Patient 9	Bleeding	Yes	6 months following the second treatment. The patient had a temporary bleeding response of 4 weeks following the first treatment.
Patient 10	Bleeding	Yes	7 months.
Patient 11	Pain, Bleeding, Mucus/Diarrhea	Yes	3-month lasting response for pain and bleeding. Significant reduction in diarrhea and mucus. The patient is no longer taking medication for this.
Patient 12	Pain	Unk	The patient reported improvement in pain.
Patient 13	Pain, Bleeding, Discharge	Yes	2-month response for all symptoms.
Patient 14	Pain, Tenesmus, Constipation	Yes	2-month response for all symptoms.
Patient 15	Bleeding, Pain	Yes	2-month response for all symptoms.
Patient 16	Bleeding, Pain, Incontinence	Yes	2-month response for bleeding and pain. Still experiencing incontinence, likely a symptom of underlying Multiple Sclerosis.

**Table 3:** Tumor response and number of treatments per patient.

Patient #	Treatment #	Weeks Since Previous Treatment	Presenting Symptoms	Tumor Size	Number of EndoVE Applications	Tumor Response
Patient 1	1	NA	Pain, Constipation	Not recorded	Not recorded	Reduction in tumor size
	2	3.5	Asymptomatic	Not recorded	Not recorded	Reduction in tumor size
	3	16	Asymptomatic	Not recorded	Not recorded	Reduction in tumor size
Patient 2	1	NA	Pain, Bleeding	Not recorded	Not recorded	Did not return for an endoscopic assessment
Patient 3	1	NA	Change in Movements, Pain, Bleeding, Anemia, Fatigue	7 cm–8 cm	12	~50% reduction in size and vascularization 2 months after initial treatment
	2	10	Asymptomatic	~4 cm	Not recorded	Unk—increase in tumor size at next visit
	3	15.5	Bleeding	8 cm × 10 cm	Not recorded	Symptomatic response to bleeding
	4	16	Obstruction	10 cm × 12 cm	8	Unk—the patient passed away 2 months later. Cause of death unrelated to disease
Patient 4	1	NA	Constipation, Pain, Bleeding	Not recorded	Not recorded	Symptomatic response for pain, bleeding, and constipation
	2	10	Asymptomatic	Not recorded	9	Transient cessation of bleeding (6 weeks). Decrease in tumor size
	3	16	Bleeding	5 cm × 15 cm	2	Decrease in tumor size
	4	23	Bleeding	5 cm × 5 cm	2	Plateau in response—stable disease
	5	18	Bleeding	5 cm × 5 cm	2	Endoscopic assessment pending
Patient 5	1	NA	Anemia Requiring Multiple Blood Transfusions and Iron Infusions	18 cm	Not recorded	Decrease in tumor size
	2	11	Asymptomatic	6 cm	4	Decrease in tumor size
	3	6.5	Asymptomatic	4 cm × 4 cm	7	Plateau in response—stable disease
	4	7.5	Asymptomatic	4 cm × 4 cm	4	Endoscopic assessment pending
Patient 6	1	NA	Anemia, Pain	6 cm × 5 cm	5	Did not return for endoscopic assessment
Patient 7	1	NA	Pain, Bleeding	6 cm	4	Complete response
Patient 8	1	NA	Change in Bowels, Loose Stools, Bloating, Flatulence	8 cm	Not Recorded	~50% reduction in tumor size
Patient 9	1	NA	Bleeding	8 cm	4	Increase in tumor size. Bulky and covering the circumference of the colon
	2	14	Asymptomatic	10 cm × 10 cm	4	>50% reduction in tumor size
	3	7.5	Asymptomatic	4 cm × 4 cm	4	Endoscopic assessment pending
Patient 10	1	NA	Bleeding	9 cm	7	~20% tumor response
	2	5.5	Asymptomatic	Not recorded	Not Recorded	Plateau in response—stable disease
	3	8.5	Asymptomatic	6 cm	4	Endoscopic assessment pending
Patient 11	1	NA	Pain, Bleeding, Mucus/Diarrhea	8 × 10	5	50% of the treated area responded
	2	5	Mucus/Diarrhea	5 cm	8	Endoscopic assessment pending
Patient 12	1	NA	Pain	2 cm × 4 cm	2	Did not return for endoscopic assessment
Patient 13	1	NA	Pain, Bleeding, Discharge	Unable to assess—extensive gastric disease extending to the lumen	2	Did not return for endoscopic assessment
Patient 14	1	NA	Pain, Tenesmus, Constipation	10 cm × 8 cm	2	Stable disease—the patient has opted for surgical intervention
Patient 15	1	NA	Bleeding, Pain	8 cm × 8 cm	5	Stable disease
	2	5	Bleeding	8 cm × 8 cm	8	Endoscopic assessment pending
Patient 16	1	NA	Bleeding, Pain, Incontinence	10 cm × 8 cm	7	~25% reduction in width of tumor

## Data Availability

The data presented in this study are available on request from the corresponding author due to patient confidentiality and GDPR.

## Abbreviations

The following abbreviations are used in this manuscript:

AE	Adverse Event
APC	Argon Plasma Coagulation
ASA	American Society of Anesthesiologists
BSG	British Society of Gastroenterology
Ca-EP	Calcium Electroporation
CCI	Charlson Comorbidity Index
CE	Conformité Europénne
CRC	Complex Colorectal Cancer
ECOG	Eastern Cooperative Oncology Group
GI	Gastrointestinal
IV	Intravenous
MDD	Medical Device Directive
MDT	Multidisciplinary Team
NHS	National Health Service
OS	Overall survival
PFS	Performance Status
QOL	Quality of Life
SAE	Serious Adverse Event
SELCA	South East London Cancer Alliance
UK	United Kingdom
